# Suppression of YAP safeguards human naïve pluripotency

**DOI:** 10.1242/dev.200988

**Published:** 2022-12-21

**Authors:** Anish Dattani, Tao Huang, Corin Liddle, Austin Smith, Ge Guo

**Affiliations:** ^1^ Living Systems Institute, University of Exeter, Stocker Road, Exeter EX4 4QD, UK; ^2^ Bioimaging Centre, Department of Biosciences, University of Exeter, Stocker Road, Exeter EX4 4QD, UK

**Keywords:** YAP, Human embryo, Pluripotency, Self-renewal, Stem cell, Trophectoderm

## Abstract

Propagation of human naïve pluripotent stem cells (nPSCs) relies on the inhibition of MEK/ERK signalling. However, MEK/ERK inhibition also promotes differentiation into trophectoderm (TE). Therefore, robust self-renewal requires suppression of TE fate. Tankyrase inhibition using XAV939 has been shown to stabilise human nPSCs and is implicated in TE suppression. Here, we dissect the mechanism of this effect. Tankyrase inhibition is known to block canonical Wnt/β-catenin signalling. However, we show that nPSCs depleted of β-catenin remain dependent on XAV939. Rather than inhibiting Wnt, we found that XAV939 prevents TE induction by reducing activation of YAP, a co-factor of TE-inducing TEAD transcription factors. Tankyrase inhibition stabilises angiomotin, which limits nuclear accumulation of YAP. Upon deletion of angiomotin-family members *AMOT* and *AMOTL2,* nuclear YAP increases and XAV939 fails to prevent TE induction. Expression of constitutively active YAP similarly precipitates TE differentiation. Conversely, nPSCs lacking *YAP1* or its paralog *TAZ* (*WWTR1*) resist TE differentiation and self-renewal efficiently without XAV939. These findings explain the distinct requirement for tankyrase inhibition in human but not in mouse nPSCs and highlight the pivotal role of YAP activity in human naïve pluripotency and TE differentiation.

This article has an associated ‘The people behind the papers’ interview.

## Teaser

**Highlighted Article:** Tankyrase inhibition suppresses trophectoderm induction to stabilise human naïve pluripotent stem cells. This effect is mediated by stabilising angiomotin to restrict YAP activity.

## INTRODUCTION

Pluripotent stem cells (PSCs) are a unique resource for developmental biology and a powerful system for biomedical research. Pluripotent stem cell counterparts of naïve epiblast in the pre-implantation embryo were first established from mice (Evans and Kaufman, 1981; Martin, 1981). Mouse embryonic stem cells (ESCs) can be propagated in a highly homogeneous state in defined media comprising inhibitors of the mitogen-activated protein kinase (ERK1 and ERK2) pathway and of glycogen synthase kinase 3 (GSK3), together with the cytokine leukaemia inhibitory factor (LIF) – a formula termed 2iLIF (Ying et al., 2008)*.* However, 2iLIF proved insufficient to capture human naïve PSCs (nPSCs). Two derivative culture conditions were developed that, in combination with mouse feeder cells, support human nPSCs with transcriptome proximity to the pre-implantation epiblast (Takashima et al., 2014; Theunissen et al., 2014)*.* The initial formulae (5iLA and t2iLGö) contained the MEK/ERK inhibitor PD0325901 (PD03) and the GSK3 inhibitor CHIR99021, plus additional kinase inhibitors and LIF. Subsequently, it was shown that human nPSCs could be established and robustly expanded without GSK3 inhibition using PD03 and LIF with the aPKC inhibitor Gö6983 and the tankyrase inhibitor XAV939 – a condition termed PXGL (Bredenkamp et al., 2019b; Guo et al., 2017).

The difference in self-renewal requirements for mouse ESCs and human nPSCs may be related to their differing lineage potency. For both mouse ESCs and human nPSCs, MEK/ERK inhibition sustains a naïve state by impeding progression to the formative stage of pluripotency (Kalkan et al., 2019; Kunath et al., 2007; Rostovskaya et al., 2019; Smith, 2017). However, unlike mouse ESCs, human nPSCs can also differentiate into trophectoderm (TE) (Guo et al., 2021; Io et al., 2021): the first extraembryonic lineage in the mammalian embryo. Remarkably, inhibition of MEK/ERK directly promotes TE induction. This effect must be countermanded to sustain human nPSCs. We have previously noted that XAV939 suppresses TE, but the mechanism is unknown (Guo et al., 2021).

XAV939 was originally identified as a WNT pathway inhibitor (Huang et al., 2009). Specifically, it is a selective inhibitor of tankyrase 1 and tankyrase 2. Tankyrases add poly-ADP-ribose to proteins, leading to elimination by the ubiquitin proteasome pathway (Smith et al., 1998). A prominent tankyrase substrate is AXIN, the scaffold protein in the β-catenin destruction complex. Tankyrase inhibition stabilises AXIN, thus promoting degradation of β-catenin, the central effector of canonical Wnt signalling (Huang et al., 2009). Tankyrases have other targets, however, including angiomotin (AMOT) (Bhardwaj et al., 2017; Wang et al., 2015). AMOT proteins are components of the HIPPO/YAP pathway. They promote the kinase activity of LATS1 and LATS2, and attenuate nuclear translocation of YAP proteins [YAP1 and its paralogue TAZ (WWTR1)] (Hirate et al., 2013; Zhao et al., 2011). By stabilising AMOTs, tankyrase inhibition can reduce YAP activity (Troilo et al., 2016; Wang et al., 2015). Notably, HIPPO and the AMOT/YAP/TEAD axis are instrumental in the segregation of TE and inner cell mass (ICM) in the early mouse embryo (Hirate et al., 2013; Nishioka et al., 2009), a process in which canonical WNT signalling has not been implicated. Here, we examine the actors downstream of XAV939 in human nPSC maintenance and TE differentiation.

## RESULTS AND DISCUSSION

### Deletion of β-catenin does not alter naïve PSC dependency on tankyrase inhibition

**Fig. 1.**
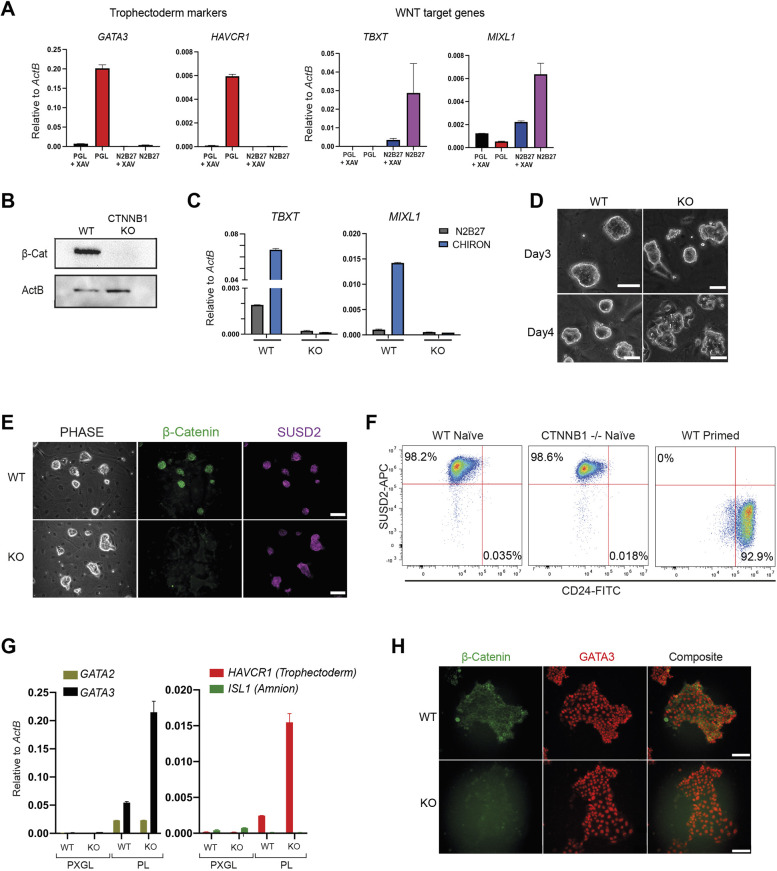
**Deletion of β-catenin does not alter naïve PSC dependency on tankyrase inhibition.** (A) qRT-PCR assays on nPSCs cultured with or without XAV939 for 3 days in PGL (PD03, Gö6983 and LIF) or N2B27. (B) β-Catenin immunoblot on wild-type and *CTNNB1* KO nPSCs expanded from a single colony. (C) qRT-PCR for canonical Wnt targets after 24 h in N2B27 with or without CHIR99021. (D) Phase-contrast images of wild-type and *CTNNB1* KO nPSCs 3 and 4 days after passage in PXGL. Scale bars: 50 µm. (E) Phase-contrast and immunofluorescence images of wild-type and *CTNNB1* KO cells in PXGL. Scale bars: 100 µm. (F) Flow cytometry analysis for surface markers SUSD2 and CD24. Primed cells were generated by capacitation of the wild-type naïve cells (Rostovskaya et al., 2019). (G) qRT-PCR for common and for TE- and amnion-specific markers after 3 days in PL. (H) Immunofluorescence images of cells in PL for 3 days. Scale bars: 100 µm. Data are mean±s.d. of PCR duplicates.

Tankyrase inhibition is commonly deployed to prevent primitive streak-like differentiation during propagation of pluripotent stem cells corresponding to post-implantation formative or primed epiblast (Kim et al., 2013; Kinoshita et al., 2021; Kojima et al., 2014; Sumi et al., 2013; Tsakiridis et al., 2014). However, for human nPSCs, the contribution of XAV939 is primarily related to suppression of TE. Withdrawal of XAV939 from PXGL self-renewal conditions leads to expression of the TE markers *GATA3* and *HAVCR1* (Guo et al., 2021). The Wnt/β-catenin targets *TBXT* and *MIXL1* are upregulated only when cells are simultaneously released from MEK/ERK inhibition (Fig. 1A, Fig. S1A). To determine whether β-catenin is relevant for the effect of tankyrase inhibition in nPSCs, we mutated the coding gene *CTNNB1.* Using Cas9 expressing HNES1-*GATA3:mKO2* reporter cells (hereafter HNES1-*GATA3:mKO2*/Cas9), we established a pool of cells that mostly lacked β-catenin protein (Fig. S1B,C). We picked and expanded a knockout clone (*CTNNB1* KO) with undetectable β-catenin (Fig. 1B, Fig. S1D). Addition of CHIR99021 failed to elicit upregulation of Wnt target genes *TBXT* and *MIXL1* in *CTNNB1* KO cells, confirming functional inactivation of the Wnt/β-catenin pathway (Fig. 1C). *CTNNB1* KO cells formed dome-shaped colonies like parental cells, although if passaging was delayed for longer than 3 days, cells began to disassemble (Fig. 1D). This may reflect a deficiency in cell-cell adhesion, as for *Ctnnb1* KO mouse ESCs (Lyashenko et al., 2011; Wray et al., 2011)*.* With a standard 3-day passaging regimen, *CTNNB1* KO cells could be stably expanded in PXGL without accumulation of differentiated cells. They maintained naïve marker expression (Fig. S1E) and displayed the cell surface marker phenotype SUSD2^+^/CD24^−^ that discriminates naïve from primed hPSCs (Bredenkamp et al., 2019a) (Fig. 1E,F).

When *CTNNB1* KO cells were plated in TE-inductive conditions of PD03 and LIF (PL) without XAV (Guo et al., 2021), they upregulated the early TE markers *GATA2*, *GATA3* and *HAVCR1* (Fig. 1G,H). Specificity of TE lineage induction was supported by the absence of the amnion marker *ISL1* (Fig. 1G) (Zheng et al., 2019). Interestingly, *CTNNB1* KO cells in PL readily formed *GATA3*-positive TE cysts (Fig. S1F). Abundant expression of plakoglobin presumably compensates for β-catenin in adherens junctions and supports epithelial integrity during TE differentiation (Haegel et al., 1995; Lyashenko et al., 2011) (Fig. S1F,G).

These findings establish that elimination of canonical Wnt signalling does not have a major influence on human nPSC self-renewal, and that absence of β-catenin does not impede nPSC to TE differentiation. Tankyrase inhibition therefore acts independently of β-catenin destruction to suppress TE fate and sustain nPSC self-renewal.

### XAV939 withdrawal leads to decreased AMOTL2 and upregulation of YAP1/TAZ targets

**Fig. 2.**
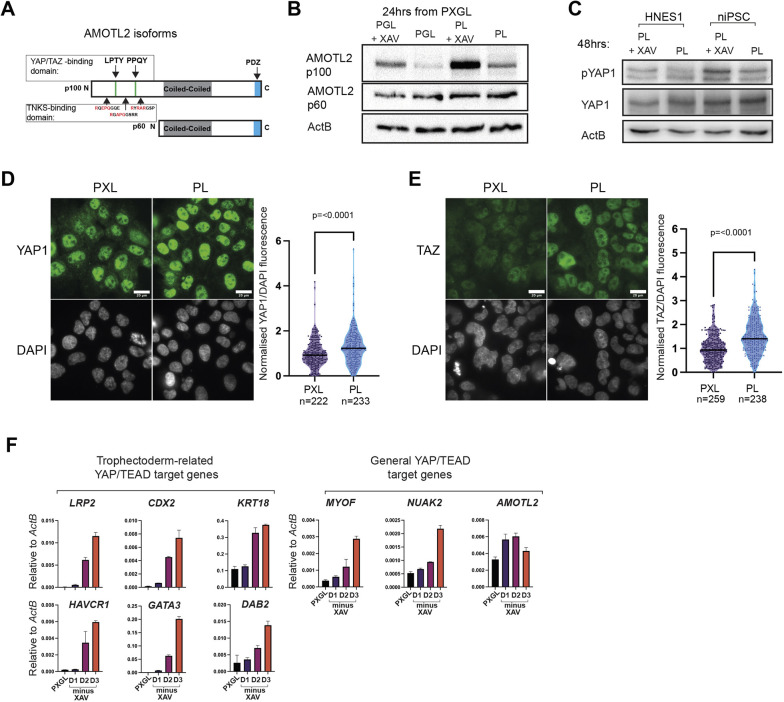
**XAV939 withdrawal leads to reduced AMOTL2 protein and upregulation of YAP targets.** (A) Schematic of AMOTL2 long (p100) and short (p60) isoforms with YAP1/TAZ, TNKs and PDZ-binding domains. (B) Western blot for AMOTL2 after transfer from PXGL to indicated conditions (see also Fig. S2B). (C) Western blot for phosphorylated YAP1 (S127) and total YAP1 after XAV withdrawal in HNES1 and niPSCs. (D,E) Immunofluorescence staining and quantification of YAP1 (D) and TAZ (WWTR1) (E) 24 h after XAV withdrawal from PXL. YAP1/DAPI or TAZ/DAPI fluorescence intensity ratios were calculated for individual nuclei in PXL and PL cultures, and plotted normalised to the mean ratio for PXL cultures. Violin plots with medians indicated by a solid bar. Two-sided Mann–Whitney *U*-test (*P*<0.0001). Scale bar: 20 µm. (F) qRT-PCR in indicated conditions for YAP/TEAD targets upregulated in mouse trophectoderm (Posfai et al., 2017) and general YAP/TEAD targets identified in various cancers (Wang et al., 2018). Data are mean±s.d. of PCR duplicates.

In immortalised cells, tankyrase inhibition has been shown to stabilise AMOT proteins, leading to reduction of YAP nuclear localisation and diminution of YAP/TEAD transcriptional activity (Troilo et al., 2016; Wang et al., 2015). The AMOT family has three members: AMOT, AMOT-like 1 (AMOTL1) and AMOT-like 2 (AMOTL2). In the early mouse embryo, AMOT and AMOTL2 prevent YAP nuclear localisation and disable TEAD-mediated transcription of TE genes in the ICM (Hirate et al., 2013; Leung and Zernicka-Goetz, 2013). Of the three paralogues, AMOTL2 mRNA is most abundant in nPSCs and also in the ICM and naïve epiblast of the human embryo (Fig. S2A,B). By western blotting, we readily detected AMOTL2 isoforms (Fig. 2A,B, Fig. S2C,D) although not AMOT. The long isoform of AMOTL2 (p100; gene accession number Q9Y2J4) contains both YAP and tankyrase binding domains in the N-terminus, and has been shown to be the target of poly-ADP-ribosylation (Cox et al., 2015; Mojallal et al., 2014; Wang et al., 2015) (Fig. 2A). On plating without XAV, we observed reduced levels of AMOTL2 p100 by 24 h, whereas expression of the shorter isoform (AMOTL2 p60; gene accession number AAH11454) was not significantly changed (Fig. 2B, Fig. S2C). Reduction in AMOTL2 protein was sustained for at least 3 days (Fig. S2D).

AMOTs regulate YAP nuclear localisation through two mechanisms: sequestration by direct binding or phosphorylation by LATS kinases, leading to degradation. In the mouse ICM, AMOT is required for phosphorylation of YAP1 and nuclear YAP exclusion (Hirate and Sasaki, 2014; Hirate et al., 2013). In nPSCs cultured without XAV, we observed a reduction in the phosphorylation of YAP1 at Ser127 (Fig. 2C). We then examined YAP1 and TAZ protein localisation by immunofluorescence staining. YAP1 exhibits heterogeneous nucleocytoplasmic staining in nPSCs (Fig. 2D). After XAV withdrawal for 24 h, we saw a moderate but significant increase in nuclear staining (Fig. 2D, Fig. S2E). This effect was more pronounced for TAZ (Fig. 2E, Fig. S2F). By 3 days, strong nuclear YAP staining colocalised with GATA3 in differentiating TE cells (Fig. S2G).

In the nucleus, YAP acts as a co-factor for TEAD transcription factors (Vassilev et al., 2001). YAP/TEAD target genes have been identified in human cancers (Wang et al., 2018) as well as in mouse trophectoderm development (Posfai et al., 2017). We surveyed a panel of YAP/TEAD targets and found they were upregulated in nPSCs from 24 h of XAV withdrawal (Fig. 2F, Fig. S2H). These findings indicate that withdrawal of tankyrase inhibition in nPSCs leads to increased nuclear YAP and activation of a TEAD transcriptional program.

### XAV inhibition of TE induction is mediated by AMOT proteins

**Fig. 3.**
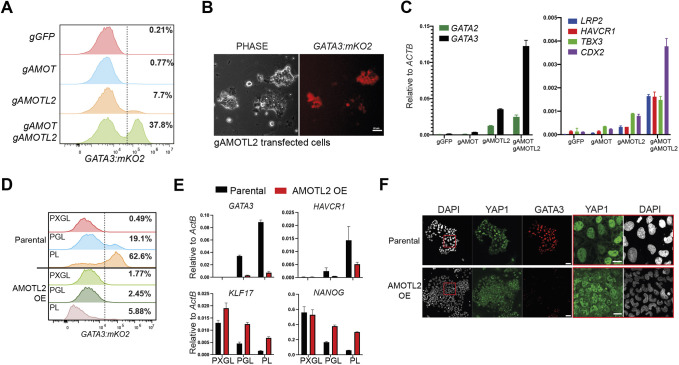
**XAV inhibition of TE induction is mediated by AMOT proteins.** (A) Flow cytometry analysis for *GATA3:mKO2* in PXGL 5 days after indicated gRNA transfection and puromycin selection. (B) Phase and fluorescence images of *AMOTL2* gRNA transfected cells in PXGL. Scale bar: 50 µm. (C) qRT-PCR for TE markers after transfection with indicated gRNAs and culture in PXGL for 5 days. Data are mean±s.d. of PCR duplicates. (D) Flow cytometry analysis for *GATA3:mKO2* in parental and *AMOTL2*-overexpressing (OE) cells in indicated conditions for 3 days. (E) qRT-PCR marker analysis in indicated conditions after 3 days. Data are mean±s.d. of PCR duplicates. (F) Immunofluorescence for YAP1 and GATA3 after 3 days in PL. Scale bars: 50 µm. The areas outlined in red are shown in the two right-most images. Scale bars: 20 µm.

We tested whether the effect of tankyrase inhibition on TE induction requires AMOT. We mutated the three *AMOT* genes individually or in pairwise combinations. Five days after gRNA transfection, we assessed *GATA3:mKO2* expression in PXGL. Flow cytometry analysis detected around 7% of cells expressing *GATA3:mKO2* in the *AMOTL2* gRNA transfected pool (Fig. 3A,B). Co-transfection of *AMOT* and *AMOTL2* gRNAs yielded more than 30% positive cells (Fig. 3A). AMOTL1 gRNA transfection had a negligible effect (Fig. S3A). Western blotting confirmed reduced AMOTL2 protein in knockout pools (Fig. S3B). The expression of *GATA3:mKO2* was accompanied by the upregulation of TE markers, increased nuclear YAP1 staining and the expression of YAP/TEAD targets (Fig. 3C, Fig. S3C). These results indicate that AMOT function is required for XAV939-mediated suppression of TE. Notably, however, in the absence of PD03, combined *AMOT* and *AMOTL2* depletion had only a marginal effect on GATA3 expression (Fig. S3D), confirming the crucial contribution of MEK/ERK to TE induction from human nPSCs. Furthermore, AMOT and AMOTL2 double depletion did not increase TE induction by PD03 (Fig. S3E).

We then investigated whether overexpression of *AMOTL2* could counteract PD03-induced TE differentiation. We introduced a CAG-*AMOTL2* expression construct into HNES1-*GATA3:mKO2* cells (Fig. S3F). Stable transfectants showed greatly diminished upregulation of *GATA3:mKO2* or TE markers on withdrawal of XAV939 and maintained expression of pluripotency genes (Fig. 3D,E). YAP1 protein remained heterogeneously distributed between cytoplasm and nucleus in PL (Fig. 3F). These results establish that AMOT proteins are necessary and sufficient for the effect of tankyrase inhibition in suppressing TE differentiation.

### Depletion of YAP1 enables sustained self-renewal without XAV939

**Fig. 4.**
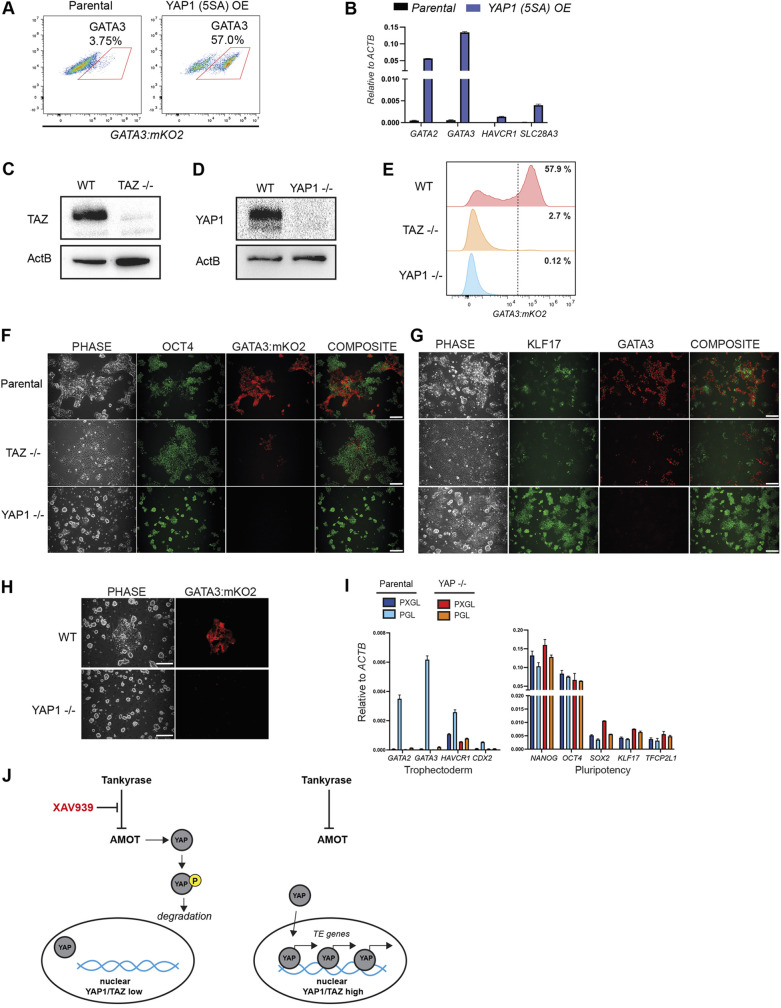
**Depletion of YAP1 abolishes TE differentiation and enables stable self-renewal without XAV.** (A) Flow cytometry for *GATA3:mKO2* in PXGL 4 days after transfection with a YAP1 (5SA) expression vector. (B) qRT-PCR for TE markers in parental versus YAP1 (5SA) transfected cells in PXGL (5 days). (C,D) Immunoblots for TAZ (C) or YAP1 (D) in wild-type and expanded *TAZ* or *YAP1* knockout cells. (E) Flow cytometry for *GATA3:mKO2* expression after 4 days in PL. (F,G) Immunofluorescence for OCT4 and *GATA3:mKO2* (F) and for KLF17 and GATA3 (G) in parental, *YAP1*^−/−^ and *TAZ*^−/−^ cells after 4 days in PL. Scale bars: 100 µm. (H) Parental HNES1 *GATA3:mKO2* and *YAP1^−/−^* cultures after 10 passages in PGL. Scale bars: 250 µm. (I) qRT-PCR assays in parental and *YAP1^−/−^* cultures after 10 passages in either PXGL or PGL. Data are mean±s.d. of PCR duplicates. (J) Schematic depicting the mechanism by which XAV stabilises human naïve pluripotency and restricts trophectoderm cell fate.

We tested whether YAP regulates the naïve to TE transition by expressing an active form of YAP1 (5SA) that cannot be phosphorylated by LATS proteins (Zhao et al., 2007). YAP1 5SA expression led to a marked upregulation of *GATA3:mKO2* reporter expression and TE genes in PXGL (Fig. 4A,B, Fig. S4A,B). Thus, constitutive YAP activity overrides the ability of tankyrase inhibition to suppress TE.

We then deleted *YAP1* or its paralog *TAZ*. Pools of cells transfected with either *YAP1* or *TAZ* gRNA showed markedly reduced frequency of *GATA3:mKO2* induction when transferred to PD03 (Fig. S4C). Co-transfection with *YAP1* and *TAZ* gRNAs completely abolished *GATA3:mKO2* expression. qRT-PCR confirmed reduced TE marker expression in *YAP1* gRNA and *TAZ* gRNA single transfectants, and in *YAP1* plus *TAZ* co-depleted cells (Fig. S4D).

We picked and expanded *YAP1* KO and *TAZ* KO cells. Interestingly, double-KO cells did not expand, suggesting that some level of active YAP is required in nPSCs. Western blotting confirmed absence of YAP1 and TAZ proteins in the respective single KOs (Fig. 4C,D). When plated in PL, *YAP1* KO cells showed minimal induction of *GATA3:mKO2* (Fig. 4E) and retained expression of OCT4 and the naïve pluripotency factor KLF17 (Fig. 4F,G). The phenotype was less strong in *TAZ* KO cells, which exhibited reduced KLF17, but these cells also largely failed to upregulate GATA3:mKO2(Fig. 4F,G).

We tested whether *YAP1* knockout enables efficient self-renewal without the requirement for XAV. We plated parental and *YAP1 KO* cells in PGL without XAV, and cultured for more than 10 passages. In the parental cultures, areas of *GATA3:mKO2*-positive cells appeared after 3-4 days at each passage (Fig. 4H). In contrast, *GATA3:mKO2*-positive cells were rarely observed in *YAP1* KO cultures. *YAP1* KO cells maintained naïve markers and showed minimal expression of trophectoderm genes or YAP1/TAZ-TEAD targets (Fig. 4I). We also mutated *YAP1* in naïve induced pluripotent stem cells (niPSCs) generated by RNA-mediated reprogramming. These independent *YAP1* KO cells similarly displayed robust self-renewal without a requirement for XAV (Fig. S4C,D). In summary, these results demonstrate that YAP activation mediates human nPSC to TE differentiation and the effect of XAV939 is to suppress this signal (Fig. 4J).

Our findings clarify the apparent contradiction between the self-renewal requirements of mouse naïve ESCs for GSK3 inhibition, which stabilises β-catenin, and of human nPSCs for tankyrase inhibition, which destabilises β-catenin. In mouse, β-catenin prevents the repressor Tcf7l1 (Tcf3) from destabilising naive pluripotency (Hoffman et al., 2013; Martello et al., 2012; Wray et al., 2011). Human nPSCs, however, barely express *TCF7L1* or its key target *ESRRB* (Boroviak et al., 2018; Rostovskaya et al., 2019). Thus, the presence of β-catenin is not important for human nPSC self-renewal. Consistent with this, deletion of β-catenin does not alter nPSC dependency on XAV939, refuting the suggestion that degradation of β-catenin mediates human nPSC propagation (Bayerl et al., 2021). Instead, the relevant effect of tankyrase inhibition is to stabilise AMOTL2 and reduce YAP1/TAZ nuclear activity. YAP regulation is crucial because of the capacity of human nPSCs for TE differentiation: a fate that is closed to mouse ESCs.

nPSCs offer new potential for dissecting mechanisms of early human embryogenesis. YAP has a well-established role in the first lineage segregation in mouse morulae (Nishioka et al., 2009). YAP nuclear accumulation in outer cells promotes TEAD-dependent transcription of TE genes, while in inner cells the AMOT complex restricts YAP to the cytoplasm (Hirate et al., 2013; Leung and Zernicka-Goetz, 2013). Our study indicates that the key role of YAP in TE differentiation is conserved between mammals, corroborating recent comparative embryology studies (Gerri et al., 2020). A second component of PXGL [and its forerunner t2iLGö (Takashima et al., 2014)], the aPKC inhibitor Gö6983, further buffers human nPSCs against TE differentiation (Guo et al., 2021). aPKC inhibition blocks the establishment of apicobasal polarity, which is implicated in initiation of TE differentiation in mouse and human (Gerri et al., 2020).

Two additional features are noteworthy. First, although AMOT depletion results in TE induction it only does so if MEK/ERK is inhibited. Future investigations will reveal how MEK/ERK inhibition enables TE lineage specification while simultaneously suppressing the formative transition. Second, we have been unable to expand nPSCs doubly deficient for *YAP1* and *TAZ*, suggesting that some degree of activity of these transcription co-factors is required for human nPSC propagation. It will be interesting to delineate the specific function of YAP in self-renewal and to understand how expression levels or dynamics of nuclear YAP direct distinct outcomes.

## MATERIALS AND METHODS

### Cell culture

Human nPSCs HNES1-*GATA3:mKO2/*Cas9 (Guo et al., 2021) and niPSCs (Bredenkamp et al., 2019b) have been published previously. Cells were maintained without antibiotics and regularly tested negative for mycoplasma by PCR.

Human naive PSCs were propagated in PXGL medium containing 1 μM PD0325901, 2 μM XAV939, 2 μM Gö6983 and 10 ng/ml human LIF (L) on irradiated or mitomycin-inactivated MEF feeders as described previously (Bredenkamp et al., 2019b). Cultures were passaged by dissociation with Accutase (Biolegend, 423201) every 3 to 5 days. Rho-associated kinase inhibitor (Y-27632) and Geltrex (0.5 μl per cm^2^ surface area; hESC-Qualified, Thermo Fisher Scientific, A1413302) were added during replating.

### Differentiation assays

nPSCs were plated in PXGL with Y-27632 on Geltrex coated plates. The day after plating, cultures were washed with PBS and transferred to N2B27 with appropriate inhibitors or cytokines for the particular assay. Concentrations of inhibitors and cytokines used were: 1 μM PD0325901, 2 μM XAV939, 2 μM Gö6983 and 10 ng/ml human LIF (L). Medium was refreshed every day thereafter.

### Gene knockout in Cas9 expressing naive cells

gRNA oligos (Table S1) were annealed to double-stranded DNA and cloned into a *Piggybac* (*PB*) vector (CML32) with a U6 promoter. CML32 contains a puromycin resistance gene and a T2A-BFP gene (Guo et al., 2021). gRNA-expression plasmids were transfected together with *PBase* plasmid into HNES1*-GATA3:mKO2/Cas9* cells (Guo et al., 2021) using the Neon Transfection system (Invitrogen). After transfection, cells were plated onto Geltrex-coated plates in PXGL with Y-27632 for 1 day before exchanging to culture media relevant for the assay, except in Fig. S3D where cells were plated directly into PXL or N2B27 after Neon transfection. Puromycin (0.5 μg/ml) was then applied for at least 3 days to select cells with *PB-gRNA* plasmid integration.

### Gene knockout by gRNA/Cas9 protein complex transfection

Trueguide synthetic gRNAs were purchased from Thermo Fisher Scientific. gRNAs were reconstituted in TE buffer to 10 μM stock. For transfection, 1 μl of 10 μM oligo was incubated with 1500 ng of TrueCut Cas9 Protein V2 for 20 min at room temperature in resuspension buffer according to the manufacturer's instructions to form gRNA/Cas9 protein complex (RNP). The RNP protein complex was mixed with dissociated nPSCs (about 2×10^5^) and transfection performed using the Neon system.

### AMOTL2 and YAP1 (5SA) transgene expression

The AMOTL2 long isoform open reading frame was amplified by PCR from total cDNA. Myc-YAP1(5SA) was amplified from pQCXIH-Myc-YAP-5SA (Addgene 33093, deposited by Kun-Liang Guan, University of California San Diego, USA) (Zhao et al., 2007)**.** PCR products were cloned into a TOPO pENTR/D-TOPO vector and the insert confirmed by Sanger sequencing. The cDNA was Gateway cloned into a PiggyBac vector behind a CAG promoter. The PiggyBac vector contains a PGK-hygromycin cassette. *PB-CAG-AMOTL2* or *PB-CAG-Myc-YAP1-5SA* plasmid was transfected into *GATA3:mKO2/Cas9* reporter cells together with *PBase* plasmid using the Neon system. Hygromycin selection was applied for 24 h to establish stable transgenic cell lines. *PB-CAG-Myc-YAP1-5SA* transfectants cells were assayed 5 days after transfection.

### Reverse transcription and real-time PCR

Total RNA was extracted using a ReliaPrep kit (Promega, Z6012) and cDNA synthesised with GoScript reverse transcriptase (Promega, A5004) and 3′Race (oligo dT) adaptor primers. TaqMan assays (Thermo Fisher Scientific) and Universal Probe Library (UPL) probes (Roche Molecular Systems) were used to perform gene quantification. UPL primers and Taqman assays are listed in Table S1.

### Western blotting

Cells were scraped from adherent cultures and lysed with RIPA lysis buffer [150 mM NaCl, 50 mM Tris (pH 8.0), 1% Triton x-100, 0.5% deoxycholate, 1 mM EDTA and 0.1% SDS] supplemented with benzonase, phosphatase and protease inhibitors. Protein concentrations were determined using a 660 nm Pierce protein assay. More than 5 μg of protein were mixed with DTT (10 mM final) in sample loading buffer and heat-denatured before separation on a 10% SDS-PAGE gel. After electrophoresis, proteins were transferred onto a nitrocellulose membrane and blocked with 5% bovine serum albumin in Tris–buffered saline containing Tween-20 (0.1%) (TBST) for 1 h at room temperature. Primary antibodies were diluted in blocking solution and incubated with the membrane overnight at 4°C or for 2 h at room temperature. After washing with TBST, the membrane was incubated with species-specific HRP-conjugated antibodies diluted (1:1000) in blocking solution. A Novex ECL Chemiluminescent substrate reagent kit was used for developing the membrane and imaging was carried out on the BioRad GelDoc XR+ system.

### Immunofluorescence staining of adherent cells

Adherent cells were washed twice with PBS and fixed in 4% formaldehyde for 10 min. Cells were permeabilised with 0.3% Triton-X/PBS solution for 15 min, and subsequently incubated with 5% BSA+0.1% Triton-X/PBS blocking solution for 1 h at room temperature. Samples were incubated with primary antibodies (Table S1) at 1:500-1:1000 dilution in blocking solution either for 1-2 h at room temperature or overnight at 4°C. After washing three times for 15 min in 0.1% Triton-X/PBS solution, AlexaFluor-conjugated secondary antibodies (Table S1) were applied at 1:1000 in blocking solution. Cells were washed in 0.1% Triton-X/PBS at least three times for 15 min before imaging. Imaging was performed on a Leica DMI-8 or Zeiss LSM880 in Airyscan mode.

### Quantification of YAP1/TAZ nuclei fluorescence intensities

Cells were plated in Ibidi polymer-coverslip chamber slides in PXL (Gö6983 was omitted to allow cell spreading and facilitate imaging) for 24 h and media were then renewed or changed to PL for 24 h. Cells were fixed and immunostained for YAP1 or TAZ, using Alexa Fluor 488 secondary antibody (Table S1). Nuclei were stained with DAPI. Images were acquired using Zeiss Elyra 7 operated in highly inclined and laminated optical sheet mode (HILO) equipped with a C-Apochromat 40×/1.2 Korr FCS M27 immersion objective. At least 20 fields of view were randomly selected. Stacks were subset and maximum intensity projections (MIPs) were performed in Zen Blue 3.3. MIPs were imported into Imaris v9.9.1 and background subtraction performed for both DAPI and 488 (YAP/TAZ) channels. Nuclei were segmented with the DAPI channels, and fluorescence intensities were extracted for both channels. Ratios of 488 (YAP1/TAZ) to DAPI intensities were calculated for individual nuclei and normalised to the mean ratio of the control PXL condition.

### Flow cytometry analysis

For β-catenin staining, cells were dissociated with TrypLE, washed twice in PBS and pelleted by centrifugation at 300 ***g*** for 5 min. Cells were then fixed in 4% formaldehyde solution for 15 min on a rotator mixer and washed twice with PBS. Cells were blocked in 2% foetal bovine serum (FBS)/PBS for 1 h at room temperature and incubated with primary antibodies (Table S1) for 1 h in blocking solution. Cells were washed three times with PBS and incubated with AlexaFluor 488 antibodies (Table S1) in blocking solution for 1 h at room temperature. Cells were washed three times and resuspended in PBS for flow cytometry analysis.

For surface marker SUSD2 or CD24 staining, live cells were dissociated with TrypLE, washed and incubated with fluorescence-conjugated primary antibodies (Table S1) diluted in PBS with 2% FBS for 1 h at 4°C. Cells were washed and resuspended in PBS before analysis.

Flow cytometry was carried out on a CytoFlex cytometer (Beckman Coulter). Flow data were analysed using FlowJo software. TOPRO3 was used to exclude dead cells.

## Supplementary Material

10.1242/develop.200988_sup1 Supplementary information
