# Supplementary material for: Suppression of YAP safeguards human naïve pluripotency
Source: Development. 2022 Dec 21;149(24):dev200988. doi: 10.1242/dev.200988 (PMC9845734; doi:10.1242/dev.200988)
Supplement: Supplementary information [file develop-149-200988-s1.pdf]

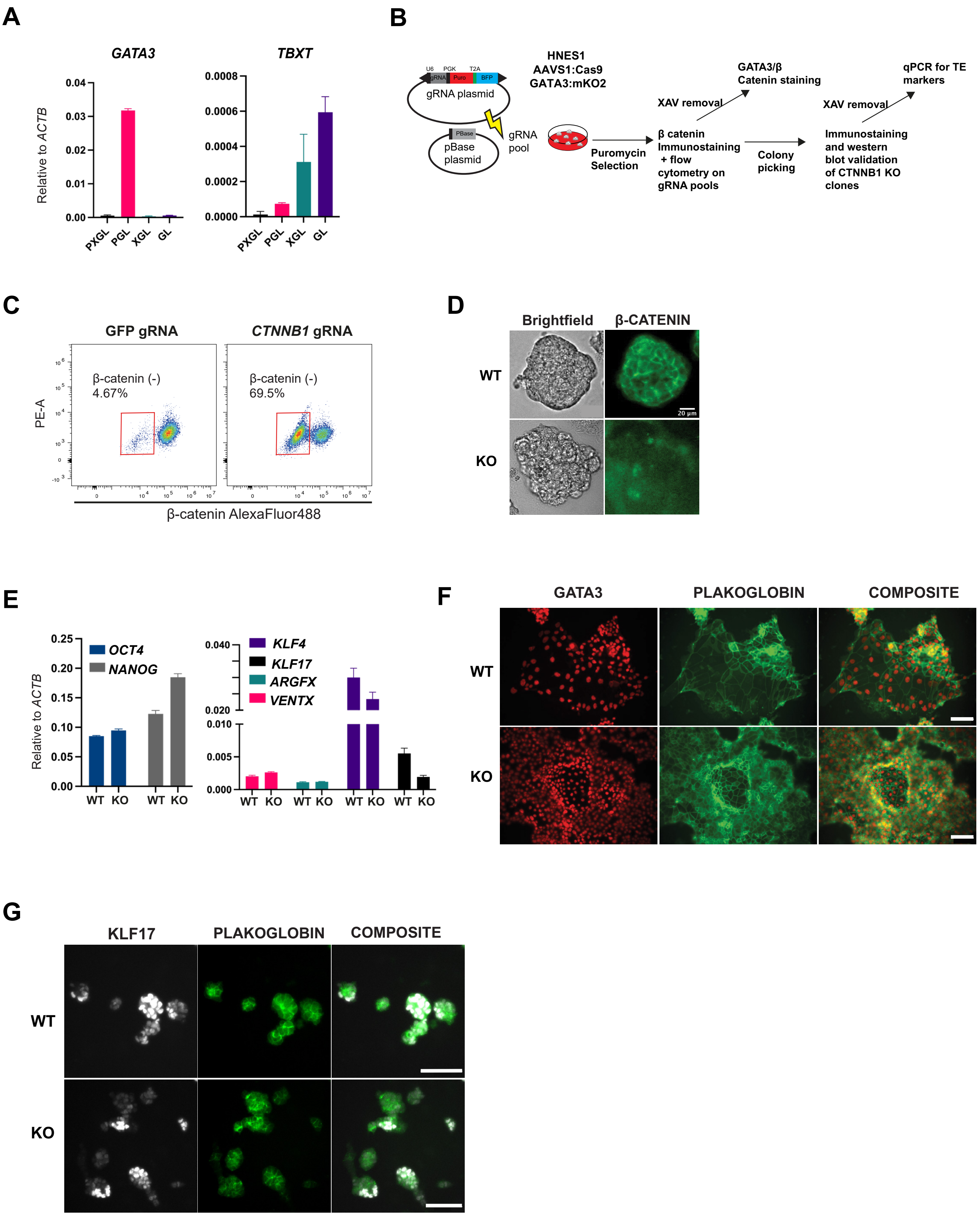

Development • Supplementary information

Fig. S1. A. qRT-PCR for TE marker *GATA3* and WNT target gene *TBXT* following 3 days transfer from PXGL to indicated culture conditions. Error bars indicate s.d. of PCR duplicates.

B. Schematic of strategy for generating and validating CTNNB1 KO human nPSCs.

C. Flow cytometry analysis following β-catenin immunostaining in GFP and *CTNNB1* gRNA transfected pools expanded for 10 passages in PXGL.

D. Immunofluorescence staining for β-catenin in wild-type and clonal CTNNB1 KO nPSCs. Scale bar: 20 µm.

E. qRT-PCR for pluripotency markers in wild-type and CTNNB1 KO cells. Error bars indicate s.d. of PCR duplicates.

F. Immunofluorescence staining for Plakoglobin and GATA3 in WT and CTNNB1 -/- cells following culture in N2B27 containing PD03 and LIF (PL) for 3 days. Scale bar: 100 µm.

G. Immunofluorescence-staining for naïve marker KLF17 and Plakoglobin in wild-type and CTNNB1 KO nPSCs cultured in PXGL on MEF. Scale bar: 100 µm.

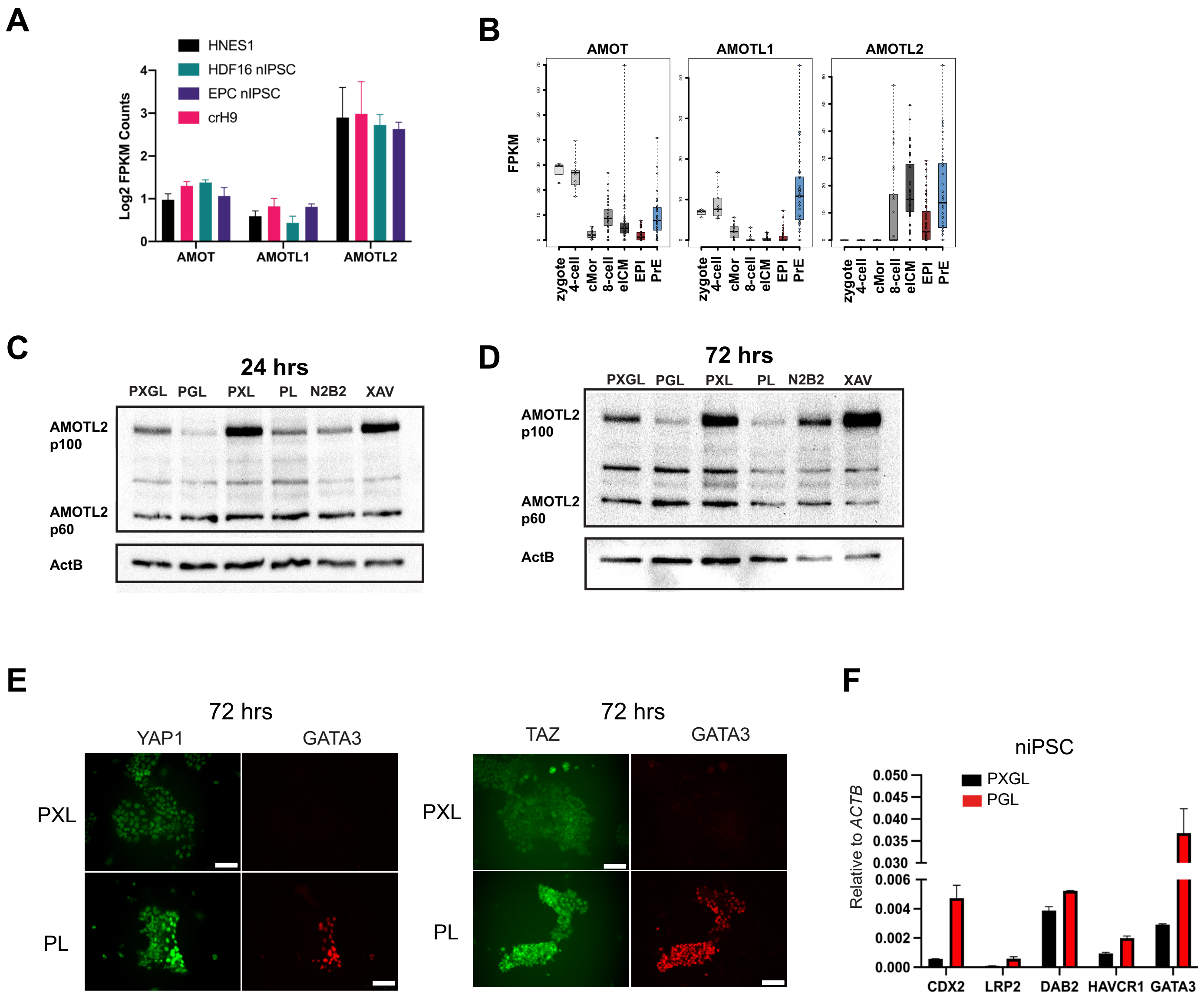

Fig. S2. A. FPKM counts for AMOT paralogs in naïve PSCs in published dataset GSE150933 (Stirparo et al., 2021).

B. FPKM expression values of AMOT family genes derived from scRNA-seq datasets for early human embryo. Graphs taken from the GRAPPA web app (Boroviak et al., 2018) and indicate the first and third quartile of expression levels. The midline identifies the median. Each dot corresponds to an individual cell.

C. Complete western blot for Fig. 2C showing AMOTL2 expression in indicated conditions for 24 hrs following transfer from PXGL.

D. Western blot showing AMOTL2 expression in indicated conditions for 72 hrs following transfer from PXGL.

E. Immunofluorescence staining of YAP1 or TAZ and GATA3 after 72 hrs in N2B27 containing PD03, XAV, LIF (PXL) or PD03 and LIF (PL). Scale bar: 100 µm.

F. Expression of YAP/TEAD target genes in niPSCs cultured in PXGL or PGL for 3 days.

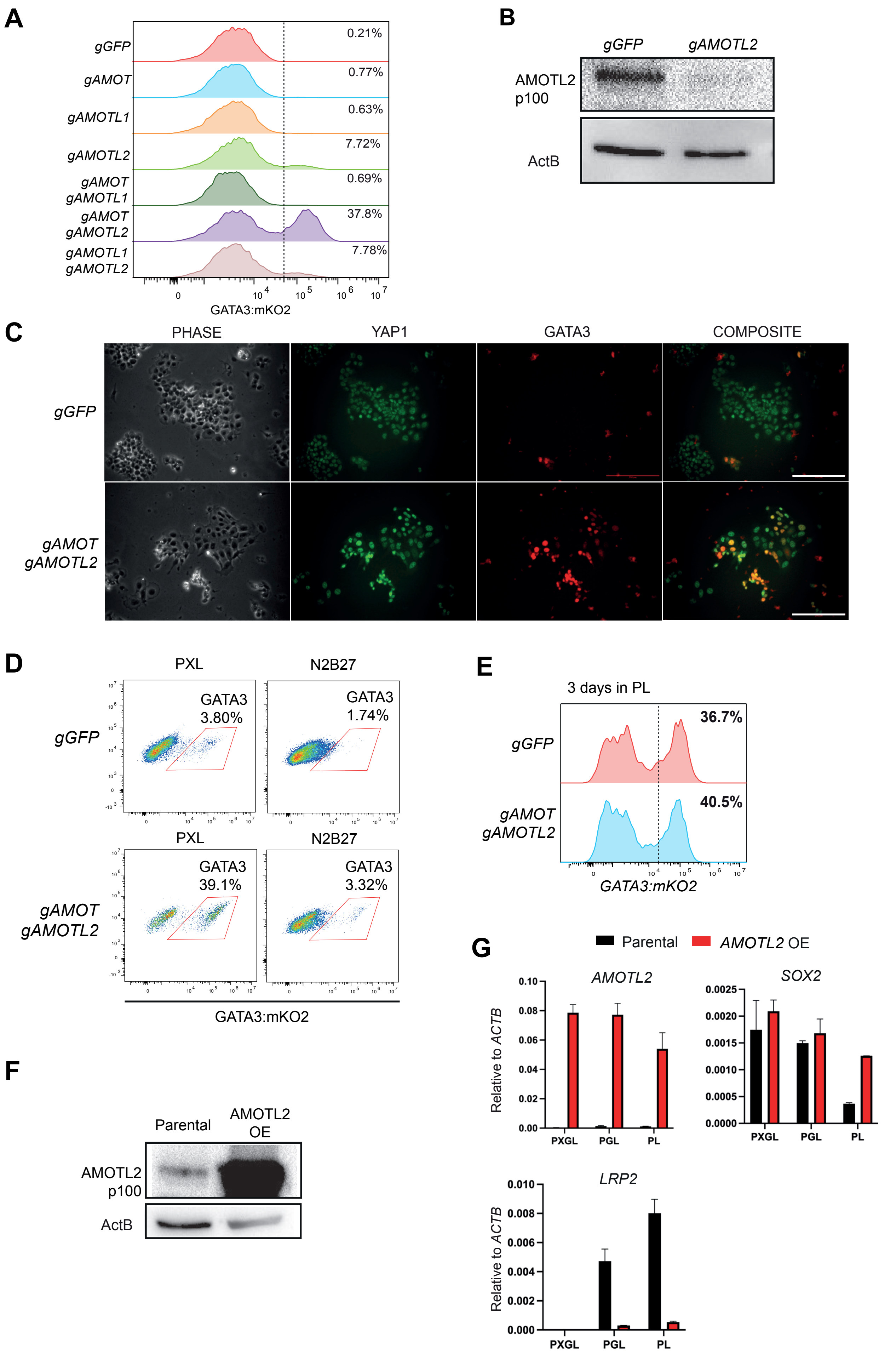

Development • Supplementary information

Fig. S3. A. Flow cytometry analysis for *GATA3:mKO2* reporter in cells transfected with indicated AMOT gRNAs. Cells were cultured in PXGL 5 days following gRNA transfection and puromycin selection.

B. Western blot of AMOTL2 six days following GFP or AMOTL2 gRNA transfection. Cells were cultured in PXGL prior to protein collection.

C. Immunofluorescence staining for YAP1 and GATA3 five days following transfection with *GFP* and *AMOT/AMOTL2* gRNAs in PXL. Scale bar: 200 µm.

D. Flow cytometry for *GATA3:mKO2* reporter expression following *AMOT/AMOTL2* double gRNA or *GFP* gRNA transfection. Cells were cultured in N2B27 or N2B27 containing PD03, XAV, LIF (PXL) for 4 days.

E. Flow cytometry for *GATA3:mKO2* reporter expression following *AMOT/AMOTL2* double gRNA or *GFP* gRNA transfection, and culture in PL for 3 days.

F. Western blot for *AMOTL2* in parental and in cells expressing AMOTL2 transgene (AMOTL2 OE).

G. qRT-PCR assays for trophectoderm and naïve pluripotency markers for parental and AMOTL2 OE cells in PL 3 days following transfer from PXGL. Data are mean± s.d. of PCR duplicates.

Development • Supplementary information

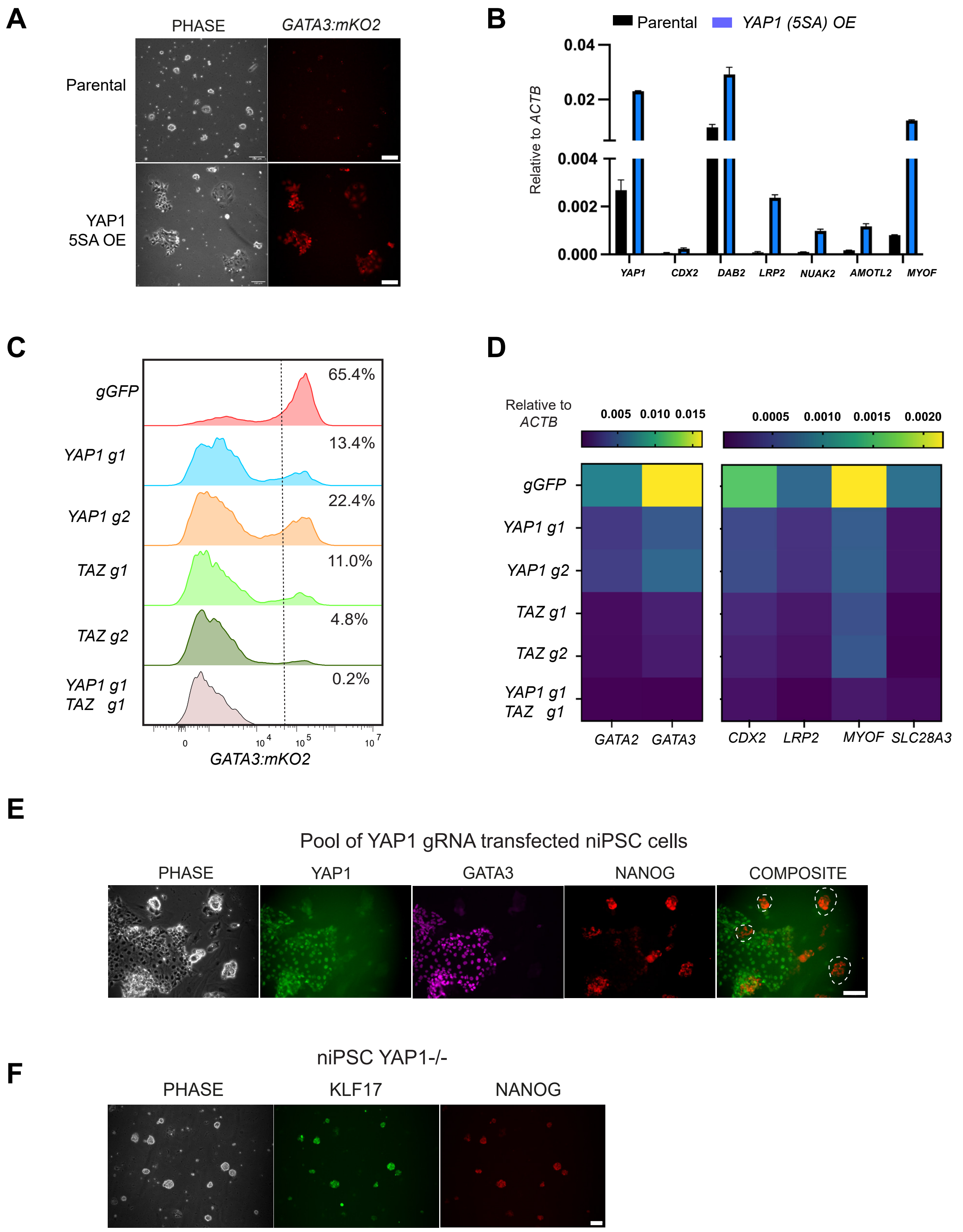

Fig. S4. A. Phase and fluorescence images of naïve cell cultures in PXGL in parental and in cells 3days after transfection with *YAP1 (5SA)* OE vectors. Scale bar: 100 µm

B. Trophectoderm and YAP-TEAD target gene expression in cells 5 days followingtransfection of *YAP1 (5SA)* OE vectors.

C. Flow cytometry analysis for *GATA3:mKO2* reporter in cells transfected with indicatedgRNAs. Cells were cultured in PD03 for 4 days.

D. Heatmap of the expression of TE markers in gRNA transfected pools as in Fig. 4A. Expression levels are means of duplicate qRT-PCR assays.

E. Immunofluorescence staining in naïve iPSCs (niPSC) after *YAP1* gRNA/Cas9 proteincomplex transfection and 4 days' culture in PL. Circles indicate *YAP1* -/- naïve-like colonies lacking both GATA3 and YAP1 expression. Scale bar: 100 µm

F. Phase and immunofluorescence images of a *YAP1* -/- niPSC clone after 4 days' culture inPL. Colonies remained dome-shaped and expressed naïve pluripotency markers KLF17 and NANOG. Scale bar: 100 µm

Development • Supplementary information

Table S1. gRNA oligoes, qRT-PCR assays and antibodies

Click here to download Table S1

Development • Supplementary information
