# Supplementary material for: Suppression of YAP safeguards human naïve pluripotency
Source: Development. 2022 Dec 21;149(24):dev200988. doi: 10.1242/dev.200988 (PMC9845734; doi:10.1242/dev.200988)
Supplement: Supplementary file 1 [file develop-149-200988_review_history.pdf]

Suppression of YAP safeguards human naïve pluripotency

Anish Dattani, Tao Huang, Corin Liddle, Austin Smith and Ge Guo
DOI: 10.1242/dev.200988

Editor: Patrick Tam

Review timeline
Original submission: 6 June 2022
Editorial decision: 29 June 2022
First revision received: 10 October 2022
Editorial decision: 1 November 2022
Second revision received: 9 November 2022
Accepted: 11 November 2022

Original submission

First decision letter

MS ID#: DEVELOP/2022/200988

MS TITLE: Suppression of YAP safeguards human naive pluripotency

AUTHORS: Anish Dattani, Tao Huang, Austin Smith, and Ge Guo

I have now received all the referees' reports on the above manuscript, and have reached a decision. The referees' comments are appended below, or you can access them online: please go to BenchPress and click on the 'Manuscripts with Decisions' queue in the Author Area.

As you will see, the referees express considerable interest in your work, but have some valid criticisms and recommend a revision of your manuscript before we can consider publication. If you are able to revise the manuscript along the lines suggested, which may involve further experiments, I will be happy receive a revised version of the manuscript. Your revised paper will be re-reviewed by one or more of the original referees, and acceptance of your manuscript will depend on your addressing satisfactorily the reviewers' major concerns. Please also note that Development will normally permit only one round of revision.

If it would be helpful, you are welcome to contact us to discuss your revision in greater detail. Please send us a point-by-point response indicating your plans for addressing the referee's comments, and we will look over this and provide further guidance.

Please attend to all of the reviewers' comments and ensure that you clearly highlight all changes made in the revised manuscript. Please avoid using 'Tracked changes' in Word files as these are lost in PDF conversion. I should be grateful if you would also provide a point-by-point response detailing how you have dealt with the points raised by the reviewers in the 'Response to Reviewers' box. If you do not agree with any of their criticisms or suggestions please explain clearly why this is so.

Reviewer 1

Advance summary and potential significance to field

Human naïve pluripotent stem cells (nPSCs) can be propagated in the presence of MEK/ERK inhibitor PD0325901, tankyrase inhibitor XAV939, aPKC inhibitor Goe6983 and LIF. XAV939 inhibits

© 2022. Published by The Company of Biologists under the terms of the Creative Commons Attribution License (https://creativecommons.org/licenses/by/4.0/).

trophectoderm (TE) differentiation promoted by the inhibition of MEK/ERK; however, the mechanisms by which XAV939 suppresses TE differentiation remained unknown.

In this study, the authors have examined the mechanism by which XAV939 suppresses differentiation of human nPSCs into TE. Although XAV939 was originally identified as a Wnt pathway inhibitor, the authors have shown that ß-catenin is not involved in the maintenance of nPSCs by XAV939. Without XAV939, the amount of AMOTL2 was reduced and the expression of YAP1/TAZ-TEAD target genes was increased. Loss of AMOT proteins promoted TE differentiation in nPSCs in the presence of XAV939, whereas overexpression of AMOTL2 suppressed the differentiation into TE in the absence of XAV939. Finally, in the absence of YAP1, human nPSCs self-renewed without XAV939. Based on these observations, the authors have concluded that YAP1 mediates TE differentiation in human nPSCs and that the major effect of XAV939 is to suppress this action.

This finding is novel and potentially important for the field. However, some points need to be clarified further. Especially, the mechanisms of regulation of YAP1/TAZ by AMOT proteins are not clear.

Comments for the author

1. Figure 2. Changes in the nuclear accumulation of YAP1/TAZ are not clearly observed in the images shown in Figures 2D–2F. Nuclear YAP1/TAZ signals should be quantified and summarized in a graph.

2. Figures 2 and S2. In the absence of XAV939, AMOT proteins were degraded and YAP1/TAZ accumulated in nuclei. Compared to the rapid degradation of AMOTL2, the nuclear accumulation of YAP1/TAZ was very slow. This raises the question whether AMOT proteins directly regulate YAP1/TAZ. It is possible that cell differentiation status also affects the nuclear accumulation of YAP1/TAZ. Please comment on this point.

3. AMOT proteins regulate YAP1/TAZ via two mechanisms: direct binding and Hippo pathway activation. In the former case, YAP1/TAZ colocalizes with AMOT proteins at tight junctions. In the latter case, the phosphorylation of YAP increases.
Figures 2E and S2C show that YAP1/TAZ did not colocalize with junctional AMOTL2, suggesting that direct binding is not the major mechanism. If YAP1/TAZ is regulated through Hippo pathway activation, the phosphorylation of YAP/TAZ-S127 should be altered.
Please examine the phosphorylation levels of YAP1/TAZ in nPSCs cultured in PL+XAV939 and PL alone.

4. Figure 2G. The selected YAP1/TAZ target genes are TE specific genes.
Their expression is regulated by differentiation status and, therefore, may not directly reflect the activity of YAP1/TAZ-TEAD. Expression of the artificial TEAD reporter (8xGTIIC-luciferase) and/or more general target genes of YAP-TEAD, e.g., AMOTL2 CTGF and CYR61, should also be examined.

(The following two comments are optional)
5. Figure 2D shows that TAZ accumulated in the nuclei preceding YAP1, and Figure 4A shows that disruption of TAZ had a stronger impact on TE differentiation than disruption of YAP1. However, the authors only examined the role of YAP1 by generating YAP1 mutant cells. Is there any reason why TAZ mutant cells were not generated?

6. Figures 3E, F, and S3D. The overexpression of AMOT proteins in cells tends to produce abnormally thick F-actin bundles in the cytoplasm. The abnormal nuclear morphologies of AMOTL2 overexpressing cells shown in Figure 3F indicate that the level of AMOTL2 was too high. Although these cells did not express TE marker genes did they maintain the expression of naïve pluripotency markers?

Minor comments
1. Line 129. “focussed” should be corrected as “focused.”
2. Line 458. Detailed reference information pertaining to this paper is missing.

© 2022. Published by The Company of Biologists under the terms of the Creative Commons Attribution License (https://creativecommons.org/licenses/by/4.0/).

Reviewer 2

Advance summary and potential significance to field

Dattani et al. investigate the mechanism of action of the tankyrase inhibitor XAV939 (XAV) in human naïve pluripotency. While this compound was originally identified as an inhibitor of the canonical Wnt pathway by stabilizing the ß-catenin destruction complex, tankyrases also have other cellular targets, such as the junction-associated Angiomotin (AMOT) protein family. Dattani et al. report that naïve human pluripotent stem cells (hPSCs) deficient in CTNNB1, the gene encoding ß-catenin, can be stably maintained in the presence of XAV and remain competent for trophectoderm (TE) differentiation upon XAV withdrawal.
These findings indicate that tankyrase inhibition acts independently of the canonical Wnt pathway to maintain naïve hPSCs and suppress TE fate. This led the authors to investigate whether XAV may act instead by stabilizing AMOT proteins which have a well-documented role in suppressing TE fate in mouse pre-implantation embryos by promoting the degradation of YAP. Indeed, removal of XAV from naïve hPSCs caused a reduction in AMOTL2 protein expression and a gradual increase in nuclear levels of YAP. To test the necessity and sufficiency of AMOT proteins in mediating the effect of XAV, the authors transfected naïve hPSCs with gRNAs targeting AMOT family members or overexpressed AMOTL2. Consistent with their hypothesis, the combined ablation of AMOT and AMOTL2 resulted in upregulation of TE markers in the presence of XAV, while the overexpression of AMOTL2 prevented GATA3 induction upon XAV withdrawal. Finally, the authors also confirmed that YAP knockout naïve hPSCs have reduced potential to differentiate into TE, a phenotype that was exacerbated by co-depletion of its transcriptional co-activator TAZ, and could maintain a stable naïve identity without XAV.

This manuscript resolves previously conflicting findings regarding the role of the canonical Wnt pathway in human naïve pluripotency. In particular, this work establishes that naïve hPSCs do not require ß-catenin, but instead rely on XAV to prevent YAP from inducing TE fate. This contrasts with the situation in mouse ESCs, where ß-catenin is required to counteract the transcriptional repressor Tcf7l1, and refutes a recent high-profile claim that ß-catenin is the principal target of tankyrase inhibition in naïve hPSCs. The experiments are well-designed and take advantage of multiple CRISPR KO lines to investigate the genetic requirements for ß-catenin, AMOT/AMOL2, and YAP/TAZ in naïve hPSC self-renewal and TE differentiation.

Comments for the author

I have several questions and suggestions to strengthen the major conclusions:
1. Fig. 1A shows that withdrawal of XAV from PXGL causes upregulation of TE markers, but induction of Wnt target genes only occurs when XAV is withdrawn from N2B27. Based on this observation, the authors argue that Wnt target genes are only upregulated when cells are also released from MEK/ERK inhibition.
However, the aPKC inhibitor Gö6983 and LIF were also withdrawn in this experiment. Are Wnt target genes induced upon omission of only the MEK inhibitor PD03 and XAV from the PXGL cocktail?

2. The manuscript mainly relies on real-time PCR to investigate the transcriptional effects of XAV withdrawal at select target genes (Fig. 2G) and the ability of YAP/TAZ KO to block TE differentiation (Fig. 4B). It would be instructive to profile the global transcriptional response by RNA-seq to ascertain whether XAV and YAP/TAZ KO exclusively suppress TE genes or impact a wider array of target genes in naïve hPSCs.

3. In Fig. 2D-F, the authors investigate the effect of XAV removal on nuclear levels of YAP/TAZ in the presence of the MEK inhibitor PD03 and LIF (PL). It is unclear why the aPKC inhibitor Gö6983 was omitted in this experiment, but included in the gene expression analysis shown in Fig. 2G. Since the purpose of this experiment is to identify events downstream of XAV939, it would make more sense to use “PGL” rather than “PL” conditions throughout these assays.

4. Fig. 3D shows a comparison of GATA3:mKO2 reporter activity in control or AMOT+AMOTL2 gRNA-transfected naïve hPSCs in either PXL or N2B27. It would be useful to include the level of GATA3 reporter activity upon XAV withdrawal (i.e. in “PL” conditions). Does depleting the AMOT complex in combination with XAV withdrawal have a synergistic effect on the extent of TE induction?

© 2022. Published by The Company of Biologists under the terms of the Creative Commons Attribution License (https://creativecommons.org/licenses/by/4.0/).

5. Supplemental Fig. 3A shows the consequences of individual and pairwise depletion of AMOT paralogs in PXGL media. Did the authors attempt to generate naïve hPSCs triply deficient for AMOT, AMOTL1, and AMOTL2 and if so, what was the effect on GATA3 reporter activity?

6. It would be helpful to include a diagram summarizing the effect of tankyrase inhibition in the maintenance of naïve hPSCs and suppression of TE differentiation via the AMOT complex and YAP/TAZ. The relationship between these genes may not be apparent to readers unfamiliar with the Hippo pathway. Such a diagram could also indicate the contrasting requirements for ß-catenin in mouse and human naïve pluripotency, which is arguably the most important takeaway from this study.

Minor points:
1. There seems to be a discrepancy in the extent of GATA3 induction in CTNNB1 KO naïve hPSCs at the RNA level (Fig. 1G) and the protein level (Fig. 1H).
Specifically, the KO cells show far more significant induction of GATA3 transcripts, but less substantial induction of GATA3 protein compared to WT cells. Please check whether this image is representative and whether it shows GATA3:mKO2 fluorescence (as indicated on line 108) or GATA3 immunostaining (as indicated in the figure legend).

2. Supplemental Fig. S3B confirms downregulation of AMOTL2 at the protein level in response to gRNA transfection. Can the authors include similar data for AMOT and AMOTL1?

3. Along similar lines, I could not find validation of reduced TAZ expression in the gRNA transfected pools shown in Fig. 4.

4. Please indicate “gRNA” below “GFP” and “AMOT/AMOTL2” in Fig. 3D to clarify that this is a genetic depletion assay, rather than an overexpression assay as shown in the next panel.

5. Abstract, line 20: the adverb “independently” would be more appropriate here

6. Results, line 141: please correct spelling of the past tense “led”

Reviewer 3

Advance summary and potential significance to field

In this study Dattani et al. reveal that inhibition of the YAP signaling pathway blocks trophectoderm induction in human naive pluripotent stem cells (hnPSC). The authors investigate the role of the tankyrase inhibitor XAV939 in maintaining the naive state. They find that the action of XAV939 is independent of beta catenin degradation and inhibition of Wnt signaling. Instead XAV939 increases the levels of other targets of tankyrase-mediated polyADP ribosylation targeted protein degradation, angiomotin and angiomotin 2. Increased angiomotin is responsible for a decreased nuclear localization of YAP, with (presumed) attendant activation of Hippo.
This study provides important new insight into the regulation of trophectoderm differentiation in hnPSC, and draws some parallels with early mouse trophectoderm development while explaining the differential response in the species to Wnt signaling in naive stem cells in the two species. The studies are clearly presented and the data in general strongly support the conclusions of the work.

Comments for the author

Specific comments:
1. Figure 1h. Are levels of GATA3 in WT and KO meant to reflect the RT-PCR in 1g? Delineate the cystic structure on the micrograph and indicate if these were seen in WT, and how frequently in both.

2. Figure 2def- it might be preferable to carry out some type of quantitative analysis of these immunostainings. The differences for TAZ are clear, less so for YAP; f shows only a single field of an isolated colony.

© 2022. Published by The Company of Biologists under the terms of the Creative Commons Attribution License (https://creativecommons.org/licenses/by/4.0/).

3. Figure 2G- a wider analysis of YAP1/TAZ TEAD targets would be more convincing.

4. Figure 3f see comment 2 above

5. L 176-what are the relative roles of AMOT2 v AMOT? Is it known whether these proteins have redundant functions not shared by AMOT1. To this point, were AMOT protein levels also reduced in the absence of XAV? Figure 3a suggests that this would have to be the case to induce GATA3, but protein data are shown only for AMOT2.

First revision

Author response to reviewers' comments

Reviewer 1 Advance Summary and Potential Significance to Field...

Human naïve pluripotent stem cells (nPSCs) can be propagated in the presence of MEK/ERK inhibitor PD0325901, tankyrase inhibitor XAV939, aPKC inhibitor Goe6983, and LIF. XAV939 inhibits trophectoderm (TE) differentiation promoted by the inhibition of MEK/ERK; however, the mechanisms by which XAV939 suppresses TE differentiation remained unknown.

In this study, the authors have examined the mechanism by which XAV939 suppresses differentiation of human nPSCs into TE. Although XAV939 was originally identified as a Wnt pathway inhibitor, the authors have shown that ß-catenin is not involved in the maintenance of nPSCs by XAV939. Without XAV939, the amount of AMOTL2 was reduced and the expression of YAP1/TAZ-TEAD target genes was increased. Loss of AMOT proteins promoted TE differentiation in nPSCs in the presence of XAV939, whereas overexpression of AMOTL2 suppressed the differentiation into TE in the absence of XAV939. Finally, in the absence of YAP1, human nPSCs self-renewed without XAV939. Based on these observations, the authors have concluded that YAP1 mediates TE differentiation in human nPSCs and that the major effect of XAV939 is to suppress this action.

This finding is novel and potentially important for the field. However, some points need to be clarified further. Especially, the mechanisms of regulation of YAP1/TAZ by AMOT proteins are not clear.

Reviewer 1 Comments for the Author...

1. Figure 2. Changes in the nuclear accumulation of YAP1/TAZ are not clearly observed in the images shown in Figures 2D–2F. Nuclear YAP1/TAZ signals should be quantified and summarized in a graph.

We now provide better resolution (40x) images of YAP1/TAZ staining with quantification of more than 200 nuclei across multiple fields (revised Figure 2D and 2E).

2. Figures 2 and S2. In the absence of XAV939, AMOT proteins were degraded and YAP1/TAZ accumulated in nuclei. Compared to the rapid degradation of AMOTL2, the nuclear accumulation of YAP1/TAZ was very slow. This raises the question whether AMOT proteins directly regulate YAP1/TAZ. It is possible that cell differentiation status also affects the nuclear accumulation of YAP1/TAZ. Please comment on this point.

We agree that in later TE differentiation the high levels of nuclear YAP1/TAZ reflect the identity of the cells. However, the quantification in Figure 2D&E shows an increase in nuclear YAP1/TAZ only 24h after XAV withdrawal and before GATA3 induction. Taken together with the biochemical and genetic evidence this supports a direct effect of AMOT reduction on nuclear accumulation of YAP1/TAZ.
We acknowledge that the increase upon XAV withdrawal detected by immunostaining is initially relatively modest, in part because the YAP1/TAZ signal is already partially nuclear in naïve PSCs in

© 2022. Published by The Company of Biologists under the terms of the Creative Commons Attribution License (https://creativecommons.org/licenses/by/4.0/).

PXGL (such heterogeneity is quite typical in cell cultures). This suggests that a threshold level and/or duration of nuclear YAP1/TAZ is critical for TE induction.

3. AMOT proteins regulate YAP1/TAZ via two mechanisms: direct binding and Hippo pathway activation. In the former case, YAP1/TAZ colocalizes with AMOT proteins at tight junctions. In the latter case, the phosphorylation of YAP increases.
Figures 2E and S2C show that YAP1/TAZ did not colocalize with junctional AMOTL2, suggesting that direct binding is not the major mechanism. If YAP1/TAZ is regulated through Hippo pathway activation, the phosphorylation of YAP/TAZ-S127 should be altered.
We appreciate the reviewer's comments and agree that phosphorylation of YAP/TAZ makes a major contribution to the effect of XAV. In the revised figure (Figure 2C) we show that pYAP1-S127 decreased following XAV withdrawal and that expression of a phosphorylation resistant form of YAP1(5SA) induced TE in the presence of XAV(revised Figure 4A&B).

4. Figure 2G. The selected YAP1/TAZ target genes are TE specific genes. Their expression is regulated by differentiation status and, therefore, may not directly reflect the activity of YAP1/TAZ-TEAD. Expression of the artificial TEAD reporter (8xGTIIC-luciferase) and/or more general target genes of YAP-TEAD, e.g., AMOTL2, CTGF, and CYR61, should also be examined.

The chosen list of YAP1/TAZ targets were identified as direct targets of YAP/TEAD during TE lineage segregation in mouse embryos (Posfai et al 2017). We did not include CTGF and CYR61 because they are not detectable in published transcriptome data of human embryos or naïve PSCs. We now include AMOTL2 in the revised figure.

(The following two comments are optional)
5. Figure 2D shows that TAZ accumulated in the nuclei preceding YAP1, and Figure 4A shows that disruption of TAZ had a stronger impact on TE differentiation than disruption of YAP1. However, the authors only examined the role of YAP1 by generating YAP1 mutant cells. Is there any reason why TAZ mutant cells were not generated?

Figure 4A (now in revised Figure S4C) assays bulk gRNA transfected cells in which the strength of the phenotype depends on the mutation efficiency of the different gRNAs. TAZ KO clone shows a similar but not stronger impact on TE differentiation (revised Figure 4E,F).

We did not investigate long term culture of TAZ KO cells because they exhibit a flattened morphology that merit a separate investigation as we now comment on in the text.

6. Figures 3E, F, and S3D. The overexpression of AMOT proteins in cells tends to produce abnormally thick F-actin bundles in the cytoplasm. The abnormal nuclear morphologies of AMOTL2 overexpressing cells shown in Figure 3F indicate that the level of AMOTL2 was too high. Although these cells did not express TE marker genes, did they maintain the expression of naïve pluripotency markers?
Yes, they maintained NANOG and KLF17 expression as shown in revised figure 3E and S3E

Minor comments
1. Line 129. "focussed" should be corrected as "focused."
Changed to "focused" to be consistent throughout the text

2. Line 458. Detailed reference information pertaining to this paper is missing.
corrected

Reviewer 2 Comments for the Author...

I have several questions and suggestions to strengthen the major conclusions:
1. Fig. 1A shows that withdrawal of XAV from PXGL causes upregulation of TE markers, but induction of Wnt target genes only occurs when XAV is withdrawn from N2B27. Based on this observation, the authors argue that Wnt target genes are only upregulated when cells are also released from MEK/ERK inhibition. However, the aPKC inhibitor Gö6983 and LIF were also withdrawn in this experiment. Are Wnt target genes induced upon omission of only the MEK inhibitor PD03 and XAV from the PXGL cocktail?

© 2022. Published by The Company of Biologists under the terms of the Creative Commons Attribution License (https://creativecommons.org/licenses/by/4.0/).

Yes, WNT target genes were also induced in N2B27 with Gö6983 and LIF. New data are included in revised figure S1A.

2. The manuscript mainly relies on real-time PCR to investigate the transcriptional effects of XAV withdrawal at select target genes (Fig. 2G) and the ability of YAP/TAZ KO to block TE differentiation (Fig. 4B). It would be instructive to profile the global transcriptional response by RNA-seq to ascertain whether XAV and YAP/TAZ KO exclusively suppress TE genes or impact a wider array of target genes in naïve hPSCs.

The focus of this study is the regulation of TE differentiation which is a unique feature of human naïve PSCs. We agree that YAP/TAZ has additional targets and functions, but that is a topic for future investigations. Adding RNA-seq to the present study would dilute the central message that tankyrase inhibition supports self-renewal by suppressing YAP/TAZ induction of TE. We respectfully point out that we have submitted the manuscript as a focused Report, not as a comprehensive Article.

3. In Fig. 2D-F, the authors investigate the effect of XAV removal on nuclear levels of YAP/TAZ in the presence of the MEK inhibitor PD03 and LIF (PL). It is unclear why the aPKC inhibitor Gö6983 was omitted in this experiment, but included in the gene expression analysis shown in Fig. 2G. Since the purpose of this experiment is to identify events downstream of XAV939, it would make more sense to use “PGL” rather than “PL” conditions throughout these assays.

The experiment was performed in these conditions for technical reasons. To determine nuclear vs cytoplasm staining in naïve PSCs in the presence of Gö6983 is highly problematic because the cells grow in densely compacted colonies. Upon Gö6983 withdrawal colonies flatten within 48 hours, which allows quantitative immunofluorescence staining.

4. Fig. 3D shows a comparison of GATA3:mKO2 reporter activity in control or AMOT+AMOTL2 gRNA-transfected naïve hPSCs in either PXL or N2B27. It would be useful to include the level of GATA3 reporter activity upon XAV withdrawal (i.e. in “PL” conditions). Does depleting the AMOT complex in combination with XAV withdrawal have a synergistic effect on the extent of TE induction?

We now include PL condition. We do not see that depletion of AMOT synergises with PD03 in TE induction (revised Figure S3E).

5. Supplemental Fig. 3A shows the consequences of individual and pairwise depletion of AMOT paralogs in PXGL media. Did the authors attempt to generate naïve hPSCs triply deficient for AMOT, AMOTL1, and AMOTL2 and if so, what was the effect on GATA3 reporter activity?

We did not attempt to generate triple knockout cells because AMOTL1 is barely expressed in early embryos nor in the naïve to TE differentiation time course and because AMOT and AMOTL2 double knockout cells exhibit pronounced TE induction in PXGL. Furthermore, triple gRNA transfection would reduce overall KO efficiency which would complicate interpretation.

6. It would be helpful to include a diagram summarizing the effect of tankyrase inhibition in the maintenance of naïve hPSCs and suppression of TE differentiation via the AMOT complex and YAP/TAZ. The relationship between these genes may not be apparent to readers unfamiliar with the Hippo pathway. Such a diagram could also indicate the contrasting requirements for ß-catenin in mouse and human naïve pluripotency, which is arguably the most important takeaway from this study.
We have added a summary diagram to illustrate the effect of XAV in revised figure 4. We appreciate the reviewer’s comment about the mouse versus human difference, but we feel this is sufficiently highlighted in the text and would over-complicate a diagram.

Minor points:
1. There seems to be a discrepancy in the extent of GATA3 induction in CTNNB1 KO naïve hPSCs at the RNA level (Fig. 1G) and the protein level (Fig. 1H).
Specifically, the KO cells show far more significant induction of GATA3 transcripts, but less substantial induction of GATA3 protein compared to WT cells. Please check whether this image is

© 2022. Published by The Company of Biologists under the terms of the Creative Commons Attribution License (https://creativecommons.org/licenses/by/4.0/).

representative and whether it shows GATA3:mKO2 fluorescence (as indicated on line 108) or GATA3 immunostaining (as indicated in the figure legend).

The image showed a TE cyst with enlarged differentiated cells which is commonly seen with the KO cells. The difference in cell size and density compared to parental cells gives a misleading impression of staining intensity. We now provide images of non-cyst regions at similar cell density in revised Fig 1H. We show a KO cell cyst is separately in Figure S1F.

The image shows GATA3 immunostaining. Text is corrected.

2. Supplemental Fig. S3B confirms downregulation of AMOTL2 at the protein level in response to gRNA transfection. Can the authors include similar data for AMOT and AMOTL1?

We failed to detect AMOT or AMOTL1 proteins by Western in non-transfected cells.

3. Along similar lines, I could not find validation of reduced TAZ expression in the gRNA transfected pools shown in Fig. 4.
TAZ western blot is shown in revised figure 4C.

4. Please indicate “gRNA” below “GFP” and “AMOT/AMOTL2” in Fig. 3D to clarify that this is a genetic depletion assay, rather than an overexpression assay as shown in the next panel.
Corrected

5. Abstract, line 20: the adverb “independently” would be more appropriate here
Corrected

6. Results, line 141: please correct spelling of the past tense “led”
Replaced with new text

Reviewer 3 Advance Summary and Potential Significance to Field...

In this study Dattani et al. reveal that inhibition of the YAP signaling pathway blocks trophectoderm induction in human naive pluripotent stem cells (hnPSC). The authors investigate the role of the tankyrase inhibitor XAV939 in maintaining the naive state. They find that the action of XAV939 is independent of beta catenin degradation and inhibition of Wnt signaling. Instead XAV939 increases the levels of other targets of tankyrase-mediated polyADP ribosylation targeted protein degradation, angiomotin and angiomotin 2. Increased angiomotin is responsible for a decreased nuclear localization of YAP, with (presumed) attendant activation of Hippo.
This study provides important new insight into the regulation of trophectoderm differentiation in hnPSC, and draws some parallels with early mouse trophectoderm development while explaining the differential response in the species to Wnt signaling in naive stem cells in the two species. The studies are clearly presented and the data in general strongly support the conclusions of the work.

Reviewer 3 Comments for the Author...

Specific comments:
1. Figure 1h. Are levels of GATA3 in WT and KO meant to reflect the RT-PCR in 1g? Delineate the cystic structure on the micrograph and indicate if these were seen in WT, and how frequently in both.
Please see response to Reviewer 2, minor point 1, which addresses this issue. We now provide more comparable images and the cystic structure is shown separately in Figure S1.

2. Figure 2def- it might be preferable to carry out some type of quantitative analysis of these immunostainings. The differences for TAZ are clear, less so for YAP; f shows only a single field of an isolated colony.
We provide better resolution images with quantitative analysis (see response to reviewer 1)

3. Figure 2G- a wider analysis of YAP1/TAZ TEAD targets would be more convincing.

© 2022. Published by The Company of Biologists under the terms of the Creative Commons Attribution License (https://creativecommons.org/licenses/by/4.0/).

We selected the most relevant YAP1/TEAD targets inferred from published mouse embryo dataset (See response to reviewer 1). As discussed in response to Reviewer 2, it is probable that YAP/TAZ have additional targets but that is beyond the scope of this Report focused on TE differentiation.

4. Figure 3f see comment 2 above. Can image at 20x and stitched to show a bigger view?
We now provide stitched images at 20x.

5. L 176-what are the relative roles of AMOT2 v AMOT? Is it known whether these proteins have redundant functions not shared by AMOT1. To this point, were AMOT protein levels also reduced in the absence of XAV? Figure 3a suggests that this would have to be the case to induce GATA3, but protein data are shown only for AMOT2.
At the transcript level AMOTL2 is more abundant and AMOTL1 is barely expressed in naïve cells or in embryos (Figure S2A,B). Therefore, we did not investigate AMOTL1 further. Unfortunately we failed to detect AMOT protein by Western blot, presumably due to low protein expression and limited antibody sensitivity.

Second decision letter

MS ID#: DEVELOP/2022/200988

MS TITLE: Suppression of YAP safeguards human naive pluripotency

AUTHORS: Anish Dattani, Tao Huang, Corin Liddle, Austin Smith, and Ge Guo

I have now received all the referees reports on the above manuscript, and have reached a decision. The referees' comments are appended below, or you can access them online: please go to BenchPress and click on the 'Manuscripts with Decisions' queue in the Author Area.

The overall evaluation is positive and we would like to publish a revised manuscript in Development, provided that the referees' suggestions for minor edits can be satisfactorily addressed. Please attend to all of the comments in your revised manuscript and detail them in your point-by-point response.

Reviewer 1

Advance summary and potential significance to field

In this paper, the authors revealed the action of the Tankyrase inhibitor XAV939 in propagation of human naïve PSCs. XAV939 suppression of TE differentiation is not via b-catenin inhibition but via inhibition of YAP by stabilization of AMOTL2. These findings are novel and important because they explain the differences in mouse PSCs and naïve human PSCs.

Comments for the author

I am satisfied with the changes made by the authors.

I found some typos, please check:
line 172. more than 30% positive cells positive.

line 264. to delineate the specific specific function of YAP

Reviewer 2

Advance summary and potential significance to field

Please see my prior report for a summary of the advance.

© 2022. Published by The Company of Biologists under the terms of the Creative Commons Attribution License (https://creativecommons.org/licenses/by/4.0/).

Comments for the author

The authors have carefully revised their manuscript in response to the reviewers' comments and this interesting study is now ready for publication in Development. I only noticed the following minor issues:

1. Line 164-165: "These findings indicate that tankyrase inhibition in nPSCs leads to increased nuclear YAP and activation of a TEAD transcriptional programme". I assume the authors meant to say that "withdrawal" of tankyrase inhibition in nPSCs leads to increased nuclear YAP and activation of a TEAD transcriptional programme"? Alternatively, they could conclude that "Tankyrase inhibition in nPSCs attenuates nuclear levels of YAP, thereby leading to diminution of TEAD co-factor availability."

2. Line 210-212: The OCT4 staining is shown in Fig. 4F, while the KLF17 staining is shown in Fig. 4G.

3. Line 221: The qRT-PCR analysis of TE markers in YAP1 KO cells is shown in Fig. 4I, not Fig. 4G.

4. The nice schematic in Fig. 4J is currently not cited in the main text. I suggest citing this schematic after the general conclusion on line 228.

5. The citation to Bayerl et al. (2021) is not listed in the References.

<u>Reviewer 3</u>

Advance summary and potential significance to field

Please see previous review.

Comments for the author

The authors have addressed all concerns of the reviewers (who were all positive about the manuscript) through revision of text and figures, and where appropriate have indicated what additional investigations are beyond the scope of the study and why.

Second revision

<u>Author response to reviewers' comments</u>

Reviewer 1 Advance summary and potential significance to field

In this paper, the authors revealed the action of the Tankyrase inhibitor XAV939 in propagation of human naïve PSCs. XAV939 suppression of TE differentiation is not via b-catenin inhibition but via inhibition of YAP by stabilization of AMOTL2. These findings are novel and important because they explain the differences in mouse PSCs and naïve human PSCs.

Reviewer 1 Comments for the author

I am satisfied with the changes made by the authors.

I found some typos, please check:
line 172. more than 30% positive cells positive.
corrected
line 264. to delineate the specific specific function of YAP
corrected

© 2022. Published by The Company of Biologists under the terms of the Creative Commons Attribution License (https://creativecommons.org/licenses/by/4.0/).

Reviewer 2 Advance summary and potential significance to field

Please see my prior report for a summary of the advance.

Reviewer 2 Comments for the author

The authors have carefully revised their manuscript in response to the reviewers' comments and this interesting study is now ready for publication in Development. I only noticed the following minor issues:

1. Line 164-165: "These findings indicate that tankyrase inhibition in nPSCs leads to increased nuclear YAP and activation of a TEAD transcriptional programme". I assume the authors meant to say that "withdrawal" of tankyrase inhibition in nPSCs leads to increased nuclear YAP and activation of a TEAD transcriptional programme"? Alternatively, they could conclude that "Tankyrase inhibition in nPSCs attenuates nuclear levels of YAP, thereby leading to diminution of TEAD co-factor availability."
Corrected. Added "withdrawal" to the sentence as suggested.
2. Line 210-212: The OCT4 staining is shown in Fig. 4F, while the KLF17 staining is shown in Fig. 4G.
Corrected
3. Line 221: The qRT-PCR analysis of TE markers in YAP1 KO cells is shown in Fig. 4I, not Fig. 4G.
Corrected
4. The nice schematic in Fig. 4J is currently not cited in the main text. I suggest citing this schematic after the general conclusion on line 228.
Corrected
5. The citation to Bayerl et al. (2021) is not listed in the References.
Corrected

Reviewer 3 Advance summary and potential significance to field

Please see previous review.

Reviewer 3 Comments for the author

The authors have addressed all concerns of the reviewers (who were all positive about the manuscript) through revision of text and figures, and where appropriate have indicated what additional investigations are beyond the scope of the study and why.

Third decision letter

MS ID#: DEVELOP/2022/200988

MS TITLE: Suppression of YAP safeguards human naive pluripotency

AUTHORS: Anish Dattani, Tao Huang, Corin Liddle, Austin Smith, and Ge Guo
ARTICLE TYPE: Research Report

I am happy to tell you that your manuscript has been accepted for publication in Development, pending our standard ethics checks.

© 2022. Published by The Company of Biologists under the terms of the Creative Commons Attribution License (https://creativecommons.org/licenses/by/4.0/).
